# How the COVID-19 Pandemic Has Impacted Daily Life? Assessing the Use of Web Resources for Recreational Activities in the Italian Adult Population

**DOI:** 10.3390/ijerph192215136

**Published:** 2022-11-16

**Authors:** Francesca Gallè, Elita Anna Sabella, Lavinia Bianco, Mario Maninchedda, Benedetta Barchielli, Fabrizio Liguori, Giovanna Da Molin, Giorgio Liguori, Giovanni Battista Orsi, Stefano Ferracuti, Christian Napoli

**Affiliations:** 1 Department of Movement Sciences and Wellbeing, University of Naples “Parthenope”, Via Medina 40, 80133 Naples, Italy; 2 Inter-University Research Centre “Population, Environment and Health”, University of Bari Aldo Moro, Piazza Cesare Battisti 1, 70121 Bari, Italy; 3 Department of Public Health and Infectious Diseases, “Sapienza” University of Rome, Piazzale Aldo Moro 5, 00185 Rome, Italy; 4 Department of Dynamic, Clinical Psychology and Health, “Sapienza” University of Rome, Via degli Apuli 1, 00185 Rome, Italy; 5 Department of Economics and Legal Studies, University of Naples “Parthenope”, Via Generale Parisi 13, 80132 Naples, Italy; 6 Department of Human Neuroscience, “Sapienza” University of Rome, Piazzale Aldo Moro 5, 00185 Rome, Italy; 7 Department of Medical Surgical Sciences and Translational Medicine, “Sapienza” University of Rome, Via di Grottarossa 1035/1039, 00189 Rome, Italy

**Keywords:** COVID-19, recreational activity, lifestyle, social media, information technology

## Abstract

Restriction measures imposed during the COVID-19 pandemic led to changes in people’s lives and behaviors. The aim of this paper is to assess the changes occurred in physical activity (PA), sleep, social and cultural activities and personal relationships of Italian adults during the pandemic and to evaluate the use of web-based resources to continue these activities. To this purpose, a cross-sectional study using a web-based questionnaire was carried out and both descriptive and regression analysis was performed. On a total of 1831 participants (61% females, age 18–93 years), the majority reported a decrease in PA, sleep, social and cultural activities and personal relationships, since the beginning of the pandemic. Sleep was notably affected by the use of new technologies. The regression analysis demonstrated that the use of web-based media for doing exercise was associated with being younger than 50 years and female; having a chronic condition was inversely related with the use of web resources for social and cultural activities and to maintain relationships; being employed was negatively related with the use of web media for continuing personal relationships and cultural activities; and being vaccinated against COVID-19 was positively associated with the use of the web for personal relationships. These findings confirm that the COVID-19 pandemic notably affected the daily life of Italian adults. Our results suggest that the use of technology to deal with these changes seems to be related with certain sociodemographic and health-related characteristics. These findings can be useful to identify those characteristics that can help people in copying with daily life modifications due to restriction measures.

## 1. Introduction

Since the beginning of its spread in the Italian territory, the novel coronavirus (SARS-CoV-2) has caused a dramatic increase in hospitalizations and deaths [1,2,3]. As of 26 October 2022, a total of 23,408,393 cases of COVID-19 and 178,753 deaths have been registered in Italy [1].

In response to this situation, the Italian government followed the indications of the World Health Organization (WHO) and implemented a series of measures aimed to control the virus transmission in the whole country, such as social distancing, the mandatory use of facial masks and the restriction of movement. After the first complete lockdown, which took place from March to May 2020, quarantine measures were imposed on individuals with COVID-19 and their contacts, and group events, such as social and recreational activities, were restricted in relation to the ongoing epidemiological situation [4,5]. These control strategies led to changes in many aspects of people’s lifestyles [6,7,8,9]. At the same time, in order to deal with the restriction measures, the lives of many people have been increasingly characterized by the use of new technologies for various purposes [10]. The following paragraph provides a brief overview on this evidence.

### 1.1. Literature Review on the COVID-19 Pandemic Effects on People’s Lives

In order to better explain the basis of our research, in this subparagraph we present, through a brief literature review, both the knowledge base and the background of the reality we examine here. Several studies performed worldwide and in Italy have analyzed the effects of lockdown and following restriction measures in different population groups, highlighting the worsening of dietary and physical activity (PA) habits, such as detrimental effects on sleep and psychological wellbeing, which had and probably will have further negative consequences on the health and wellbeing of large proportions of the populations [6,7,8,9,10,11,12,13,14,15,16]. Moreover, the COVID-19 pandemic and related restriction measures had also inevitable consequences on people’s social lives [13]. With the onset and the succession of lockdown and quarantine measures, many people changed their method or place of work and some had to suspend their work [17]. Furthermore, maintaining contacts with others, attending meetings and visiting locations in person were hindered.

Meanwhile, a substantial rise in the use of web-based technologies was observed from the beginning of the pandemic, with some groups using them more than others on the basis of their needs [10]. People are using new media, social media and apps for information, communication, shopping and workout more than in the past [18]. Students and workers have experienced a necessary engagement with technology in order to carry out their required activities [18]. The internet is also used to provide psychological first aid and necessary assistance to the elderly, in particular to compensate for the lack of human capabilities [8]. Even the American Psychological Association (APA) promoted the use of social media platforms to be connected with others to remain safe, to be informed and to reduce stress during the COVID-19 pandemic [19].

In fact, despite its potential negative properties, such as a potential addictive effect, using technologies may contribute to coping with a challenging situation such as the COVID-19 [18]. This issue is of particular importance for older individuals, whose access and use of information communication technologies commonly differs from that of the younger generations [20]. Older adults, who were highly affected by the negative consequences of COVID-19, were particularly impacted with to respect restriction measures, such as social distancing and self-isolation. In this group, reduced contact with others may have affected psychosocial wellbeing, in addition to physical conditions, more than in other population groups [21,22].

### 1.2. Aims of the Study

The present cross-sectional study was aimed to assess a sample of Italian adults regarding (i) changes that occurred in PA, sleep, relationships and social and cultural activities during the COVID-19 pandemic; (ii) the possible use of web-based resources related to these activities during the emergency; and (iii) the possible association between the use of web media to perform sociocultural activities or to maintain relationships during the pandemic, along with certain sociodemographic and health-related characteristics of participants. To these aims, information was collected through a web-based questionnaire and examined both through a descriptive analysis and a logistic regression.

### 1.3. Hypotheses and Research Questions

To the authors’ knowledge, no study has been performed to investigate the long-term effects of the COVID-19 pandemic on Italian adults’ daily life with regard to the use of new technologies as coping strategies.

In light of the available literature, we hypothesized that:

1. Besides its acknowledged effects on health-related lifestyles, the emergency period also produced notable changes in non-work-related activities, such as family and friendship relationships or participation in social and cultural activities of Italian adults; considering this, we formulated the research question on how much Italian citizens have relied on new technologies to address these changes.
2. An undefined part of the Italian adult population used web-based media as the main resource to maintain their recreational activities.
3. A different use of web resources during the pandemic to maintain these activities has occurred on the basis of the sociodemographic and health-related characteristics of individuals; specifically, we supposed that the use of web-based media as a resource for maintaining PA, socio-cultural life and relationships could be related to gender, age, engagement in a romantic relationship, having children, level of education and occupational status, having a chronic disease, having suffered from COVID-19 and being vaccinated against SARS-CoV-2.

## 2. Materials and Methods

This cross-sectional study was carried out by an on-line questionnaire during the period March–June 2022 in the adult population from Central and Southern Italy. The provision of an informed consent was required to complete the questionnaire. The protocol of the study was approved by the Institutional Board of the Inter University Research Centre “Population, environment and health” (CIRPAS), with the number 1303_2021.

### 2.1. Participants

A minimum sample size of at least 385 enrolled individuals was required to investigate the selected variables, assuming a response proportion of 50%, a 95% confidence level and a 5% margin error, as previously reported [13]. Participants were recruited by using the main means of communication (i.e., mailing lists, instant messaging, WhatsApp) and social networks (i.e., Facebook, Instagram). In order to reach a large convenience sample, the social media groups/chats of university students and the social media groups/chats belonging to reference figures from recreational, religious and cultural associations were used with the request of further sharing the questionnaire via their contacts. No payment was provided to participants. Inclusion criteria were being Italian, being at least 18 years of age and having electronic devices with an internet connection available. All the respondents expressed their informed consent to participate in the study.

### 2.2. Questionnaire

The online questionnaire was divided into two sections. The first part included questions aimed at collecting demographic characteristics and personal experience concerning COVID-19 and participants. Specifically, they were asked to refer their gender, age, level of education, domicile (own home/guest by relatives or other caregivers/offsite for study or work/guest in a nursing home), employment status (student/unemployed/worker/retired), marital status (married/separated or divorced/widowed/cohabiting or not with partner/single) and whether they had children or not; possible current health chronic condition; whether they had suffered from COVID-19 and in what form (asymptomatic or not severe/severe requiring hospitalization); and whether they have been vaccinated towards COVID-19; if not, they were asked to give their main motivation from the following options: health conditions/I’m not at risk/I’ve had the disease/I don’t trust vaccines in general/I don’t think that these vaccines are effective/I think that these vaccines could be dangerous.

The second section was focused on the habits assumed during the COVID-19 pandemic by participants. They were asked to refer their current weight and height in order to calculate their reported body mass index (BMI) and to declare whether their habitual physical activity, sleep, participation in social and cultural activities and relationships with non-cohabiting people has been maintained, decreased or increased. As for these five last variables, participants who reported maintained/increased habits were also asked to report the main method or reason (walking/exercise or sport practiced in a facility/exercise or sport practiced autonomously at home/exercise or sport practiced at home with web tutorials or trainers’ guide for physical activity; anxiety or worrying/more time spent in watching TV/more time spent in watching streaming platforms/more time spent playing on electronic devices/more time spent working on electronic devices/more time spent in working or leisure activities not on electronic devices for sleep; in person meetings/by telephone/through video-calls for social activities and relationships; and through in person visits/online visits for cultural activities). Respondents who reported maintaining activities and social and cultural relationships, especially through new communication media, were asked to report whether its use was already established before the pandemic or whether they learned to use these technologies during the pandemic.

The questionnaire was previously tested in a forty-people-pilot-study (data not published). A content validity evaluation was made by experts in the field. First, four professionals, experts in public health, epidemiology, psychology and communication technologies, respectively, designed the questionnaires based on the scientific literature. Subsequently, an external panel of experts provided a first evaluation of the tool. Furthermore, the measure of questionnaire’s comprehensibility was evaluated in a group of 40 people. This pilot group was asked to assign a rating to each question on a 7-point scale (Q: Does the following sentence make sense to you? 1: not meaningful; 7: very meaningful); a mean score > 5 per question was considered as the cut off for acceptability. For this purpose, the original questionnaire was modified: aside from the questions belonging to the standard questionnaire (SQQ), 10 additional questions (AQ) reporting grammatical and semantic errors (e.g., the use of the verb to be in place of to have, the use of general words, such as “things” in place of specific words, etc.) were included in order to guarantee answer variability. SQQ reported a mean score for each question > 6; AQ reported a mean score for each question < 2. These data confirmed that the content of the questionnaire was clear to the readers.

The reliability index for the final version of the questionnaire was assessed using Cronbach’s alpha (internal consistency coefficient). The alpha value achieved was 0.77 for the second section of the questionnaire. An alpha > 0.70 was also achieved after stratifying the sample by sex and age, showing a satisfactory level of internal consistency [23].

### 2.3. Data Analysis

A descriptive analysis was performed on the sociodemographic characteristics and behaviors of participants. Continuous variables were expressed as mean value ± standard deviation (SD), while categorical variables were reported as the number and percentage (%) of respondents.

A logistic regression was performed considering the use of web-based technologies during the pandemic for doing exercise, participating in social and cultural activities and connecting with people as dependent variables. Specifically, a value = 1 was assigned to the use of web resources, while a value = 0 was assigned to all the other possible answers. Age (expressed as 0 = lower or equal/1 = higher than the median value), gender (0 = male/1 = female), relationship status (0 = non-engaged/1 = engaged), being a parent (0 = no/1 = yes), level of education (expressed as 0 = non-graduated/1 = graduated), employment status (0 = unemployed/1 = employed), concurrent health condition or disease (0 = no/1 = yes), having had COVID-19 and COVID-19 vaccinated (0 = no/1 = yes) were considered as independent variables. The results were reported as odds ratio (OR) with a related 95% confidence interval (95% CI), assuming that an OR value of less than 1 indicates a lower likelihood of using web-based media for a certain category of individuals as defined by independent variables, while a value higher than 1 indicates a higher likelihood of using web media. The goodness of fit for each logistic regression model was expressed by Nagelkerke’s R squared (R^2^_N_).

Statistical significance was assumed at *p* < 0.05. Statistical analyses were conducted using SPSS (Statistical Package for Social Science; version 27.0; IMB SPSS; Armonk, NY, USA).

## 3. Results

A total of 1844 questionnaires were collected. Of these, 1831 were fully completed and were included in the analysis. The main characteristics of the sample are shown in Table 1.

The sample was composed mainly of females, individuals engaged in relationships and with children, with a mandatory-to-high school level education, employed and affected by a chronic disease. As for COVID-19, the slight majority of the participants contracted the disease and about 90% of the sample was vaccinated. The highest proportion of those who were non-vaccinated declared the possible danger for health related to COVID-19 vaccines as their main motivation.

The habits assumed by participants during the pandemic are reported in Table 2.

A decrease in physical activity, sleep, social and cultural activities and personal relationships was reported by the majority of the sample. The use of walking and in-person meetings or visits was reported as the main mean for maintaining/increasing physical activity, relationships and sociocultural activities. Watching TV on demand was reported as the main reason for sleep decrease. About 80% of those who reported the use of web media for their relationships was able to use them even before the pandemic.

As for the regression analysis, the results show that the use of web-based media for PA was associated with being younger than 50 years and female, being engaged in a romantic relationship and not having children; the use of the web to maintain social activities was positively related with having a partner and negatively related with having a children and a chronic condition; the use of web resources for personal relationships was negatively associated with being employed and having a chronic disease and positively with being vaccinated against COVID-19; the use of web-based technologies for cultural activities was negatively related with being employed and having a chronic disease (Table 3).

## 4. Discussion

This study aimed at exploring the changes occurred in daily life of Italian adults since the beginning of the COVID-19 pandemic, at assessing the use of web media as resources to maintain their previous habits, and to highlight the possible predictors of this behaviors. As for the first item, a general worsening of lifestyle and recreational activities during the pandemic was registered in the sample examined. In fact, a decrease of physical activity, sleep and all the domains of social relationships investigated was reported by the majority of the participants.

The results, regarding health-related behaviors, are in line with previous studies carried out in Italy that reported an increase in sedentary behavior and in time spent sitting or lying down watching television or using mobile devices and a worsening or reduction in sleep [14,24,25]. Alongside the direct effects related to decreased mobility, the reduced PA combined with increased time spent sitting may reduce the effectiveness of the feedback system that controls appetite levels, leading to an overconsumption of food and weight gain [26]. This, together with stockpiling food due to the restrictions on grocery shopping and boredom and stress due to the continuous streaming of news related to COVID-19, has led to negative changes in lifestyle [8,14]. Moreover, the reduction of PA levels was reported to be associated with a decrease in sleep quality during the pandemic [27]. In our study, the majority of respondents who decreased their sleep during the pandemic reported watching TV on demand as the main cause of reduction and another 10.6% reported more time spent on a computer. In fact, digital device usage prevents individuals from going to sleep at an optimal time, leading to sleep pattern disturbances [28], to changes in sleep–wake rhythms (people going to bed and waking up later) and to a lower sleep quality [29]. These changes could also be attributed to changes in daily schedules created by the confinement, which may contribute to poor sleep quality due to disruptions in circadian rhythms [26]. Moreover, these effects have been showed to be higher in people who were physically active before the pandemic [30].

Furthermore, a reduction in social and cultural activities, such as in personal relationships, was also reported by the majority of our sample. This is of concern, since maintaining social relationships is associated with better cognitive performance and can help in preserving cognitive functions [31,32,33,34,35].

Among those who continued these activities, the majority reported that in-person meetings and visits were the main methods they used, even during the pandemic. With regard to this aspect, it should be noted that in the study performed by Sage et al., the higher number of social contacts registered among Italian citizens largely accounted for the higher infection rates registered in the Italian context than in the German and British ones [36]. This allows to hypothesize a low adherence of Italian people to restrictive measures and requires further analyses.

However, in our sample, a proportion of respondents ranging from 22.4 to 33.7% reported the use of web as the main resource for maintaining socio-cultural activities and personal relationships. For instance, during the lockdown, people used videoconferencing services to schedule ‘happy hours’ with co-workers and friends, celebrate holidays and life milestones with loved ones, keep in contact with their previous communities (such as houses of worship, fitness studios and community centers) through streaming live services, as well as to seek out and make new connections [18]. Therefore, the use of videoconferencing may alleviate depression and loneliness and improve social support; as a consequence, those who reported the use of web-based resources for their relationships may have experienced fewer psychological side effects [18].

As for our third hypothesis, the regression analysis showed interesting relationships between sociodemographic variables and the use of web media for recreational activities during the pandemic. The use of web resources to practice exercise during the pandemic was related to younger age, female gender, being in a romantic relationship and not having children. Time spent on the internet increased during lockdown [28,37], mostly because social media was engaged directly as the fastest method for spreading information regarding the COVID-19 pandemic [8]. Moreover, internet allowed the use of a large number of mobile apps related to health and fitness focusing on meditation, exercise and nutrition, many of which have been associated with improved health in users [18]. TikTok, in particular, had a large share of participation and offered short fitness-based videos during the COVID-19 pandemic [38]. Our results confirm that females were more interested than males in exercising at home with online instructions during the pandemic period [39]. As for age and gender, another Italian study aimed at assessing the relationships among loneliness, anxiety and excessive social media use showed that women and younger adults seem to be not only more engaged in online social connections but also more interested by negative feelings and dysfunctional social media use [40].

In our sample, being engaged and not being a parent played a role on the use of web resources to carry out social activities. Moreover, being employed was negatively related with the use of web media for maintaining personal relationships and cultural activities during the pandemic. It is arguable that individuals with these sociodemographic characteristics were allowed to or preferred to maintain their recreational activities through in-person contacts rather than through web media.

Some studies have tried to analyze the effects of the pandemic on the life of employees from different countries [41,42,43]. The new arrangements made to the labor market during the COVID-19 pandemic, especially those based on flexible and remote working, have changed traditional work and work-related relationships [42]. The possibility of working from home can be a positive factor that can afford workers more freedom to spend leisure time with family and allow the reduction of commuting time, supporting their work–life balance. However, over time, these working patterns can cause an imbalance between work and life. This imbalance can lead to stress and depression, absenteeism, reduced job satisfaction and health problems [43]. Moreover, teleworkers are shown to be vulnerable to social isolation in the context of the pandemic, which highlights the possible role that the type of work can have on social life [44]. Nevertheless, the present survey did not examine in-depth the work type of employed participants (remote/on site/flexible), the possible changes that occurred in their working activity during the course of the pandemic, nor the possible related negative feelings. However, it seems that these aspects played a role in the choice of how individuals continued their daily life activities and should be further investigated.

In addition, it seems that having a chronic condition hindered the use of web resources for continuing socio-cultural activities and maintaining contact with family and friends. This may have led to social isolation. Similarly, it is well known that the great majority of those who reported social isolation had one or more chronic conditions [45]. Moreover, during the pandemic, there were reports of older people abandoned in care homes, making them more vulnerable to social isolation and loneliness, with an increased risk of anxiety, depression, cognitive dysfunction, heart disease and mortality [22]. However, to the best of our knowledge, the literature that similarly focuses on the connection between having a chronic condition and the employment of web media for personal relationships is lacking, and we are therefore unable to compare our findings with others.

Finally, having been affected by COVID-19 was not associated with any of the outcomes, while the COVID-19 vaccination was positively related with the use of web media for personal relationships. Our result might be a consequence of the fact that vaccinated individuals could have a tendency to be more careful and to keep avoiding all activities that may lead to a higher risk of contagion. This is consistent with evidence from the UK during the second wave of COVID-19, when vaccinated individuals increased compliance with behavioral measures to reduce the spread of COVID-19, such as the wearing of masks, practicing social distancing and reducing household mixing [46]. However, it must be kept in mind that, as a general rule, vaccinated individuals are reported to be less careful and have more social contacts than unvaccinated individuals, both in person and by web media [47,48].

This study has some limitations. First of all, the method of questionnaire administration may represent a selection bias, since only people who were reached by the survey and who had access to the internet was enrolled; for this reason we included reference figures from recreational, religious and cultural associations in order to try to reduce this effect on the results. Furthermore, although participants were asked to report possible changes that occurred in their behaviors during the pandemic in respect to the pre-pandemic period, it should be considered that since the beginning of the SARS-CoV-2 spread, the Italian population have experienced different epidemiological situations and related control measures. Therefore, it is possible that they gave their answers as habits assumed in a specific phase (i.e., the lockdown) rather than to settled behaviors. Moreover, our study is based on self-reported PA, a method which suffers from various limitations, although it demonstrated the association between accelerometer recorded data and reported physical activities [49].

However, to the best of our knowledge, this is the first study focusing on the employment of web media for PA and recreational activities during the COVID-19 pandemic in a large sample of the Italian population. It allows us to highlight some possible associations between sociodemographic characteristics and the use of new technologies in challenging conditions. Further research should be carried out to examine these aspects in depth.

## 5. Conclusions

Since the beginning of the COVID-19 pandemic, many studies have testified to the changes that occurred in people’s moods and lifestyles, focusing on their possible health effects. Alongside this, an increasing use of web-based media has been observed, mainly to carrying on work and study notwithstanding social distancing. In this study, we have analyzed the extent to which Italian adults have used new technologies as a resource to maintain their physical activity, sociocultural activities and personal relationships during the pandemic and the contribution of new media to their sleep worsening.

The results of the study show that, in our sample, COVID-19 notably affected PA, sleep and social life. As for sleep, it seems that the new technologies played an important role in it, during the pandemic. As for the other activities, although the majority of the participants who maintained their pre-pandemic habits continued to have in-person contacts, the use of web media as a resource for maintaining previous activities was shown to be relevant and associated with certain sociodemographic and behavioral characteristics. In particular, youths and females seem to be keener to use web-based resources for continuing PA even during the emergency, while being employed and having a chronic disease did not favor the use of these technologies for continuing social life in the course of the emergency. Moreover, people vaccinated against COVID-19 were more interested in the use of social media to maintain their personal relationships. Therefore, this study offers a picture of which groups were mostly interested in the availability of new communication technologies during the COVID-19 emergency. In our sample, female and younger individuals found in web media a resource with which to deal with restriction measures that hindered exercise outdoors or at a sporting facility and vaccinated people benefited from them to maintain their personal contacts by respecting social distancing measures, while employed individuals and those who had a chronic condition preferred to maintain in-person contacts even during the pandemic. These aspects could be useful to address future strategies aimed at supporting people when choosing the most suitable way to copy with restriction measures adopted in such emergency situations.

## Figures and Tables

**Table 1:** Characteristics of the sample.

Variable	Participants (*n* = 1831)
Age	mean ± SD
	47.7 ± 17.3
	range
	18–93 years
Gender	*n* (%)
female	1116 (61.0)
male	715 (39.0)
Romantic relationship	*n* (%)
engaged	1127 (61.6)
non-engaged	704 (38.4)
Children	*n* (%)
no	861 (47.0)
yes	970 (53.0)
Educational level	*n* (%)
mandatory to high school	955 (52.2)
degree and post-degree	876 (47.8)
Occupational status	*n* (%)
student/unemployed/retired	894 (48.8)
employed	937 (51.2)
Chronic disease	*n* (%)
no	799 (43.6)
yes	1032 (56.4)
Contracted COVID-19	*n* (%)
no	889 (48.6)
yes	942 (51.4)
non-severe form	850 (90.2)
severe form	92 (9.8)
COVEID-19 vaccination	*n* (%)
no	223 (12.2)
yes	1608 (87.8)
Reason for non-vaccination	*n* (%)
health conditions	49 (28.5)
I’m not at risk	11 (6.4)
I’ve had the disease	3 (1.7)
I don’t trust vaccines	5 (2.9)
I don’t think that these vaccines are effective	2 (1.2)
I think that these vaccines could be dangerous	102 (59.3)
No answer	51 (22.9)

**Table 2:** Habits assumed during the pandemic by respondents.

Variable	Number of Respondents (%)
Physical activity	
Unchanged	548 (29.9)
Increased	333 (18.2)
Decreased	950 (51.9)
Physical activity maintained/increased through:	
Walking	374 (42.5)
Autonomous exercise at home	227 (25.8)
Online coaching or tutorials at home	72 (8.2)
Supervised exercise in a facility	206 (23.4)
Sleep	
Unchanged	598 (32.7)
Increased	230 (12.6)
Decreased	1003 (54.8)
Sleep decreased because of:	
Anxiety or worrying	233 (23.2)
More time spent in watching TV	200 (19.9)
More time spent in watching TV on demand	303 (30.2)
More time spent on a computer	106 (10.6)
More time spent with hobbies/work	30 (3.0)
Other reasons	131 (13.1)
Social activities	
Unchanged	314 (17.1)
Increased	39 (2.1)
Decreased	1478 (80.7)
Social activities maintained/increased through:	
In-person meetings	188 (53.3)
By phone calls	37 (10.5)
By videocalls	119 (33.7)
Personal relationships	
Unchanged	798 (43.6)
Increased	151 (8.2)
Decreased	882 (48.2)
Personal relationships maintained/increased through:	
In-person meetings	591 (57.9)
By phone calls	280 (27.5)
By videocalls	149 (14.6)
Cultural activities	
Unchanged	230 (12.6)
Increased	42 (2.3)
Decreased	1559 (85.1)
Cultural activities maintained/increased through:	
In-person visits	211 (77.6)
Internet	61 (22.4)
Use of web-based media during the pandemic:	
I was able before	116 (77.9)
I learned during the pandemic	33 (22.1)

**Table 3:** Results of the regression analyses performed considering the use of web-based resources for PA, social activities, relationships and cultural activities during the pandemic as dependent variables, with related R-squared values for each model.

	Outcomes			
Independent Variables	Use of Web-Based Media for PA OR (CI 95%)	Use of Web-Based Media for Social Activities OR (CI 95%)	Use of Web-Based Media for Relationships OR (CI 95%)	Use of Web-Based Media for Cultural Activities OR (CI 95%)
Age				
≥49 years	Reference	Reference	Reference	Reference
>49 years	0.140 (0.052–0.379) *	0.839 (0.496–1.420)	0.866 (0.553–1.355)	1.418 (0.715–2.814)
Gender				
male	Reference	Reference	Reference	Reference
female	4.305 (1.912–9.693) *	1.152 (0.743–1.786)	0.827 (0.572–1.195)	0.728 (0.423–1.254)
Relationship status				
non-engaged	Reference	Reference	Reference	Reference
engaged	2.065 (1.139–3.742) *	1.581 (1.022–2.446) *	0.880 (0.608–1.273)	0.838 (0.484–1.450)
Children				
no	Reference	Reference	Reference	Reference
yes	0.482 (0.257–0.902) *	0.606 (0.376–0.977) *	0.700 (0.461–1.061)	0.598 (0.315–1.135)
Educational level				
non-graduated	Reference	Reference	Reference	Reference
graduated	0.715 (0.401–1.275)	0.833 (0.538–1.289)	0.962 (0.657–1.409)	0.924 (0.518–1.646)
Occupational status				
unemployed	Reference	Reference	Reference	Reference
employed	0.941 (0.511–1.732)	0.687 (0.437–1.080)	0.673 (0.453–1.000) *	0.436 (0.235–0.808) *
Chronic disease				
no	Reference	Reference	Reference	Reference
yes	0.601 (0.310–1.166)	0.270 (0.157–0.463) *	0.471 (0.303–0.731) *	0.397 (0.202–0.779) *
Contracted COVID-19				
no	Reference	Reference	Reference	Reference
yes	1.033 (0.627–1.700)	0.805 (0.538–1.203)	0.749 (0.520–1.079)	0.808 (0.466–1.399)
COVID-19 vaccination				
no	Reference	Reference	Reference	Reference
yes	0.928 (0.271–3.178)	2.062 (0.728–5.840)	3.981 (1.425–11.123) *	0.790 (0.333–1.873)
R^2^_N_	0.201	0.129	0.084	0.064

* significant associations shown in bold.

## Data Availability

All data presented are available upon request from the corresponding author (F.G.).
